# Effects of high dietary threonine supplementation on growth performance, health biomarkers, and intestinal histology in cyclic heat-stressed broilers

**DOI:** 10.14202/vetworld.2025.646-657

**Published:** 2025-03-18

**Authors:** Abia Khalid, Sania Bashir, Asma Kalsoom, Hafiz Faseeh Ur Rehman, Muhammad Afzal Rashid, Mansur Abdullah Sandhu, Habib Ur Rehman, Muhammad Shahbaz Yousaf

**Affiliations:** 1 Department of Physiology, University of Veterinary and Animal Sciences, Lahore, 54000, Pakistan; 2 Department of Anatomy and Histology, University of Veterinary and Animal Sciences, Lahore, 54000, Pakistan; 3 Department of Animal Nutrition, University of Veterinary and Animal Sciences, Lahore, 54000, Pakistan; 4 Department of Veterinary Biomedical Sciences, PMAS-Arid Agriculture University, Rawalpindi, 46300, Pakistan

**Keywords:** broilers, heat stress, intestinal health, meat quality, oxidative status, threonine

## Abstract

**Background and Aim:**

Heat stress (HS) negatively impacts poultry production by reducing growth performance and compromising physiological health. Nutritional strategies, particularly amino acid supplementation, are explored to mitigate these adverse effects. This study evaluates the impact of high dietary threonine supplementation on growth performance, health biomarkers, oxidative status, meat quality, and intestinal histology in cyclic HS broilers.

**Materials and Methods:**

A total of 288 1-day-old Hubbard broilers were randomly allocated to six treatment groups: Thermoneutral, HS control, and HS supplemented with 125% (HS-125), 150% (HS-150), 175% (HS-175), and 200% (HS-200) of NRC-recommended threonine. Birds in the HS groups were exposed to cyclic HS (35°C, 75% relative humidity) from day 22 to day 42. Growth performance was recorded weekly, while physiological parameters, oxidative stress markers, and jejunal histology were analyzed post-exsanguination.

**Results:**

HS significantly reduced body weight gain and feed intake, while threonine supplementation did not improve these parameters. However, liver weight, serum albumin, and cholesterol levels improved at higher threonine doses (175%–200%). Threonine also reduced serum corticosterone and malondialdehyde levels, suggesting enhanced stress resilience. Superoxide dismutase activity, an indicator of oxidative defense, improved in threonine-supplemented groups. In jejunal histology, acidic goblet cells increased, and intraepithelial lymphocyte infiltration decreased in birds supplemented with 175%–200% threonine, indicating enhanced gut integrity. Meat quality attributes, including crude protein and oxidative stability, showed minor but inconsistent variations across treatments.

**Conclusion:**

Although high dietary threonine supplementation (175%–200%) improved stress resilience by enhancing oxidative status, intestinal health, and selected physiological biomarkers in HS broilers, however, it failed to enhance growth performance. These findings suggest that while threonine supports physiological adaptations under HS, its use as a growth promoter under HS conditions may not be economically viable. Further studies are warranted to optimize amino acid balance in HS broilers for improved productivity.

## INTRODUCTION

Heat stress (HS) poses a formidable risk that can result in substantial economic losses for the poultry industry across tropical, arid, and semi-arid zones worldwide. Modern broilers possess a rapid metabolism, resulting in increased heat production and susceptibility to HS [1]. The impending threat of climate change and its precursor, global warming, has significantly raised the ambient temperature. The rise in temperature affects all living organisms, including humans, plants, and animals. Consequently, there is a growing concern regarding ongoing fluctuations in climatic conditions that have a huge negative impact on animal production [2]. For instance, birds exposed to high environmental temperatures exhibit behavioral, physiological, and immunological responses that negatively affect their productivity [3, 4].

Nutritional manipulation is a viable approach to mitigating the harmful effects of HS on poultry, and it is frequently implemented in conjunction with other strategies, such as management, environmental, and genetic interventions [5]. Dietary interventions encompass various approaches, including high feed density and dietary energy, as well as the inclusion of feed additives, such as probiotics [6], trace minerals [7], vitamins [8], prebiotics [9], and amino acids [10]. These interventions provide advantageous biological effects that mitigate the hazardous effects of HS [11]. These compounds can reduce stress, regulate growth, combat oxidative stress and inflammation, regulate the immune system, and improve gut health [12]. Threonine is the third-rate-limiting amino acid [13], following methionine and lysine, in corn-soybean-based diets for broilers [14]. It is important in gut development, intestinal integrity maintenance, and mucin production by the goblet cells [15]. In addition, threonine is an integral part of immunoglobulins, and its deficiency can impair immunoglobulin production [16]. Threonine supplementation has been shown to improve growth performance by improving feed intake, feed conversion ratio, body weight gain, intestinal health [10], immune function [16], antioxidant activity [17], and microbial populations [18] in broilers.

While previous studies have evaluated the role of threonine in normal and stressed conditions, most research has focused on its effects at low to moderate supplementation levels or in combination with other amino acids. There is limited evidence on the impact of high-dose threonine supplementation in broilers under cyclic HS, particularly concerning its effects on oxidative status, health biomarkers, and intestinal histology. Furthermore, the optimal threonine levels required to counteract the physiological stress induced by HS remain unclear.

This study aims to evaluate the effects of high dietary threonine supplementation on growth performance, health biomarkers, oxidative status, meat quality, and intestinal histology in broilers subjected to cyclic HS. Specifically, the study seeks to determine whether increased threonine levels can mitigate the adverse effects of HS and improve physiological and biochemical responses, thereby optimizing broiler health and welfare under HS conditions.

## MATERIALS AND METHODS

### Ethical approval

The Ethical Review Committee of the University of Veterinary and Animal Sciences (UVAS), Lahore, Pakistan (DR/1105/21/10/2017) duly approved and ensured adherence to all the protocols and procedures employed in this experimental study.

### Study period and location

The study was conducted for 42 days, starting from the end of August to the start of October 2021. The trial, sampling, and processing were conducted in the animal shed and research laboratory of the Department of Physiology, UVAS.

### Experimental design, husbandry, and housing

Day-old Hubbard broiler chicks (n = 288), procured from a local hatchery, were reared in an experimental poultry shed at the Department. The birds were weighed (average 41 g) upon arrival at the study site and randomly allotted to six groups (n = 48 birds/group). Each group was further divided into four replicates, with each replicate consisting of 12 birds. The groups were designated as thermoneutral group (TN) with NRC-recommended threonine (0.74%); HS group with NRC-recommended threonine; HS group (HS-125) with 125% NRC-recommended threonine; HS group (HS-150) with 150% NRC-recommended threonine; HS group (HS-175) with NRC-recommended 175% threonine; and HS group (HS-200) with NRC-recommended 200% threonine. Threonine doses were supplemented during the heat-stress phase in corn-soybean meal-based diets from day 22 to day 42. The stocking density was maintained at 10 birds/m^2^. The diet composition is presented in Table 1. Crystalline L-threonine (98.5% of Polifar Group Limited, China) was used to meet the required doses of L-threonine in the diets.

**Table 1:** Composition of grower diet.

Ingredients (g/100 g)	Grower diet (day-22 to day-42)
Corn	61.38
Soybean meal 44%	22.65
Sunflower meal	2.80
Canola meal	7.70
Vegetable oil	3.00
DCP	0.20
Limestone	1.50
Common salt	0.30
DL-Methionine	0.15
L-Lysine HCl	0.12
Vitamin premi×^1^	0.10
Mineral premi×^2^	0.10
Total	100.00
Calculated nutrients	
ME (kcal/kg)	2849
CP (%)	19.53
Threonine (%)	0.80

1 Provided vitamins per 2 kg of the feed: Vitamin A: 11,000 IU, Vitamin D3: 2,200 IU, Vitamin E: 22 IU, Choline chloride: 440 mg, Vitamin B12: 0.0132 mg, Riboflavin: 8.8 mg, Pantothenic acid: 22 mg, Ethoxyquin: 250 mg, Menadione: 2.2 mg, Pyridoxine: 4.4 mg, Folic acid: 1.1 mg, Biotin: 0.22, Thiamin: 4.4 mg

2 Supplied minerals per 2 kg of the feed: Cu: 20 mg, Zn: 120 mg, Mn: 100 mg, Fe: 100 mg, I: 0.92 mg, Ca: 180 mg *The analyzed concentration of threonine in the grower diet was 0.39% on a dry matter basis. To obtain the NRC level and supra-supplementation levels, L-threonine was added to the diets as required

The temperature and relative humidity (RH) were initially maintained at 35 ± 1°C and 65 ± 5%, respectively. Thereafter, the temperature of the shed was gradually reduced (3°C weekly) by turning the electric heaters to low heat or turning them off and monitored using installed temperature and humidity sensors until it reached 26 ± 1°C at the end of the 3^rd^ week while the RH remained at 65 ± 5%. The birds in the TN group were reared under the same conditions from week 4 until the termination of the experiment on day 42. Birds in the HS groups were exposed to a high environmental temperature, artificially induced using electric heaters, of 35 ± 1°C and 75% ± 5% RH from day 22 until day 42, with cyclic HS occurring from 8:00 to 18:00. All birds were immunized against Newcastle disease and infectious bursal disease following the standard vaccination schedule. The temperature-humidity index during HS is given in Table 2.

**Table 2:** Temperature humidity index during the period of heat stress in broilers.

Weeks	9:00 am	12:00 pm	03:00 pm	09:00 pm
Forth	87.77	88.63	89.17	81.66
Fifth	88.75	89.52	89.37	77.50
Sixth	89.29	89.31	88.74	79.38

### Productive performance

Performance parameters such as feed intake were calculated daily based on feed offered and leftovers, whereas body weight and body weight gain were determined at the end of each week [19]. The feed conversion ratio was calculated weekly using weight gain and feed intake data [20].

### Exsanguination and sample collection

After the termination of the trial, two birds from each replicate were chosen on a random basis and were killed by exsanguination to collect samples. The blood was collected in plain test tubes and kept at room temperature until the blood clot contracted. Thereafter, it was centrifuged at 4°C for 15 min at 800 × *g* to harvest serum. The serum samples were stored at −40°C until further analysis. The abdominal cavities of the birds were explored to collect the viscera. Immediately after measuring the length and weight of the small intestine, a 2-cm segment of the jejunum close to Meckel’s diverticulum was separated, and the lumen was flushed with chilled normal saline. Subsequently, samples were kept in 10% neutral buffered formalin (NBF) until processed for histology. A 3-cm-long breast muscle piece was cut parallel to the direction of the muscle fibers in each bird. The samples were then immediately frozen at −40°C for oxidative status determination.

### Viscera analysis

The viscera were carefully dissected and separated. The weights and lengths of the viscera were measured using an electronic balance and measuring tape, respectively. The viscera included the liver, heart, gizzard, immune organs (spleen and bursa), small intestine, and cecum. The weights were expressed in grams and the lengths were expressed in inches.

### Health biomarker analysis

The serum samples were thawed on ice and vortexed before analysis. The serum biomarkers, including cholesterol, triglycerides, and high-density lipoproteins, were estimated using diagnostic kits (Human Diagnostics Worldwide, Wiesbaden, GmBH, Germany) following the protocols and procedures provided in the kits. Aspartate aminotransferase (AST), alanine aminotransferase (ALT), alkaline phosphatase, creatine kinase, lactate dehydrogenase, total protein, albumin, creatinine, blood urea nitrogen, and uric acid levels were determined using kits (DiaSys Diagnostic Systems, Holzheim, Germany). Serum hormone levels, including free triiodothyronine, free thyroxine (Calbiotech, San Diego, California, USA), and corticosterone (CORT) (CORT enzyme-linked immunosorbent assay [ELISA] Kit, BT Lab, Birmingham, England), were estimated using ELISA kits. The readings were obtained using the Epoch™ microplate spectrophotometer (Biotek Instruments Inc., Winooski, USA). Globulin levels were calculated by subtracting the serum albumin concentration from the total protein concentration. Low-density lipoproteins were calculated using Friedewald’s formula [21].

Low-density lipoprotein (LDL) (mg/dL) = Total Cholesterol – High-density lipoprotein (HDL) – (Triglycerides ÷ 5).

### Oxidative status analysis

To assess the oxidative status in pectoral muscle and serum, the levels of malondialdehyde (MDA), catalase, and superoxide dismutase (SOD) were measured according to the methods previously described by Eva [22], Hadwan and Abed [23], and Li [24], respectively. The pectoral muscle was homogenized in phosphate buffer saline before analysis.

### Muscle physical characteristics and proximate analysis

Post-slaughter pectoral muscle pH at 45-min and 24-h was determined by inserting a glass electrode of a portable pH meter (PCE-228 M pH meter, Southampton, UK) into the muscle [25]. The water-holding capacity (WHC) was determined by placing a pre-weighed meat sample in a centrifuge and subjecting it to centrifugation at 500 g for 15 min at 4°C. Pre-centrifugation weight (Pre-C-Wt) and post-centrifugation weight (Post-C-Wt) were used to calculate the WHC of the samples [26].

WHC (%) = (Pre-C-Wt – Post-C-Wt/Pre-C-Wt) × 100

To determine the dry weight (method 930.15), 20 g pectoral muscle was precisely weighed and placed in an oven at 105°C for 16 h or till the uniform weight was achieved [27]. Thereafter, samples were frozen at –40°C until analysis. At the time of analysis, the samples were ground into small pieces and subjected to different protocols. The crude protein was determined (method 968.06) using a Nitrogen and Protein Analyzer (rapid N exceed, Elementar, Langenselbold, Germany), which employs high-temperature combustion to release and detect nitrogen from the sample. The protein factor 6.25 was used to calculate the protein content [27]. The crude fat was determined (method 920.39) using a Soxhlet apparatus with 2 g of the sample [27]. This defatted and dried sample was processed to determine (method 942.05) the ash contents. Samples were ashed at 600°C for 2 h in a muffle furnace to determine ash contents [27].

### Jejunal histology and selected cells counting

Jejunal samples, stored in 10% NBF, were processed for histology. After washing, the samples were subjected to sequential dehydration at different ethanol concentrations. After dehydration, the samples were embedded in liquid paraffin for sectioning at 5 μm thickness. After mounting the micro-sections of the jejunum on slides and de-waxing, the sections were stained with different stains as required. The stained tissues were examined using a bright-field microscope (Labomed Inc., California, USA). The morphometry program (Prog Res®2.7.7 Capture Prog Camera Control Software, Jenoptik Optical Systems, Jena, Germany) was used to determine the parameters.

Hematoxylin and eosin staining was performed to measure the histological attributes of the jejunum, including villus height (VH), villus width (VW), and crypt depth (CD), as well as for the counting of intraepithelial lymphocytes (IELs). For histological analysis, images of well-oriented villi were taken at 40×. The VH was measured from the tip to the base of the villus. VW was taken from the middle length of the villus. The CD was determined by measuring the distance from the base of the crypt to the area where the crypt transitioned into the villus. The villus surface area (VSA) was calculated using a standard formula [28].

VSA = (2 × 3.14) × (Villus width ÷ 2) × (Villus length)

For the goblet cell count, tissue sections were stained with Alcian Blue and Periodic Acid-Schiff reagents following the standard protocol. The goblet cells were counted along the entire length of the villus. Acidic and basic goblet cells were identified based on the color of the mucin present in these differentiated cells as blue or magenta, respectively [29].

For IEL counting, the middle 200 μm area of the villus was marked, and cells were counted on both sides of the villus. Images were taken at 400× to count cells. The cellular density per 100 μm was determined. The IELs were identified in the epithelial lining of the villus, which has a round nucleus and scanty cytoplasm [29].

### Chemicals

All chemicals used in the study were of analytical grade and were sourced from Merck KGaA, Darmstadt, Germany, otherwise specified.

### Statistical analysis

All data were analyzed using IBM SPSS 20 software (IBM Corp., NY, USA). Before statistical analysis, the Kolmogorov-Smirnov test was applied to assess the normality of data distribution, while Levene’s test was used to confirm homogeneity of variance. A one-way analysis of variance (ANOVA) was conducted to compare treatment means among different experimental groups. Polynomial contrast analysis (linear and quadratic) was performed to evaluate the dose-dependent effects of dietary threonine supplementation. Tukey’s *post hoc* test was applied to determine significant differences between groups when ANOVA results indicated significance. Data are expressed as mean ± standard error of the pooled means, and statistical significance was set at p < 0.05.

## RESULTS

### Productive performance

The results showed that body weight gain (BWG) was lower (p = 0.001) in the HS-125, HS-150, and HS-175 birds during the 5^th^ week compared with the TN birds, but it remained non-significantly lower in the HS and HS-200 birds compared with the TN birds. The BWG, however, during the 6^th^ week (p = 0.003) as well as during the entire period (p = 0.005) of HS (4–6 weeks) was lower in the HS and all the threonine-supplemented HS broilers compared with the TN broilers (Table 3).

**Table 3:** Effects of dietary threonine inclusion on body weight gain and feed intake of heat-stressed broilers.

Treatment groups	Weeks (body weight gain, g)				Weeks (feed intake, g)			
	
	4	5	6	4–6	4	5	6	4–6
TN	313	499^a^	646^a^	1457^a^	825^a^	1027^a^	1043^a^	2894^a^
HS	286	420^ab^	300^b^	1006^b^	759^ab^	847^ab^	899^ab^	2505^ab^
HS-125	309	322^b^	218^b^	778^b^	781^ab^	745^b^	747^b^	2274^b^
HS-150	351	288^b^	238^b^	877^b^	745^ab^	768^b^	723^b^	2237^b^
HS-175	320	271^b^	222^b^	813^b^	719^ab^	689^b^	759^b^	2167^b^
HS-200	310	365^ab^	243^b^	918^b^	618^b^	773^b^	798^b^	2189^b^
SEM	11.55	20.67	40.86	62.60	19.71	30.20	30.09	71.35
p-value	0.778	0.001	0.003	0.005	0.037	0.007	0.004	0.007
Linear	0.605	0.001	0.001	0.003	0.002	0.002	0.001	0.001
Quadratic	0.639	0.001	0.005	0.004	0.326	0.013	0.003	0.037

Data are presented as Means ± SEM. ^a-b^Different superscripts in a column differ significantly. TN=Thermoneutral, HS=Heat stress, SEM=Standard error of the pooled means.

The FI was lower (p = 0.037) in the HS-200 group than in the TN group during the 4^th^ week. However, the FI was not significantly lower in all other experimental groups than in the TN group. Moreover, FI was lower in threonine-supplemented HS birds compared with TN birds during the fifth (p = 0.007) and sixth (p = 0.004) periods as well as during the entire HS period (p = 0.007). FI in the HS broilers was non-significantly lower compared with the TN group during the HS period (Table 3). The feed conversion ratio (FCR) did not differ among the treatment groups (data not shown).

### Viscera development

Birds exposed to HS had increased (p < 0.001) liver weight compared with TN birds; however, threonine supplementation during HS reduced (p < 0.001) the liver weight. The heart weight was lower (p = 0.001) in the threonine-supplemented HS broilers than in the TN broilers, except for the HS and HS-200 broilers. Furthermore, the cecal weight was higher (p = 0.026) in the HS birds compared to the TN birds. The weights of the gizzard, spleen, bursa, and small intestine remained unchanged in all experimental groups. Similarly, the small intestinal and cecal lengths did not differ among the treatment groups (Table 4).

**Table 4:** Effects of dietary threonine inclusion on viscera weights and lengths of heat-stressed broilers.

Organs	Treatment groups									

	TN	HS	HS-125	HS-150	HS-175	HS-200	SEM	p-value	Linear	Quadratic
Weight (g)										
Liver	45.42^b^	57.93^a^	37.67^b^	45.57^b^	38.52^b^	49.01^ab^	1.44	<0.001	0.158	0.090
Heart	12.57^a^	11.79^ab^	8.26^c^	9.69^bc^	9.65^bc^	10.83^abc^	0.335	0.001	0.020	<0.001
Pancreas	3.50	3.84	3.51	3.16	3.57	4.21	0.10	0.080	0.249	0.053
Gizzard	27.74	30.48	26.89	29.79	28.36	29.65	0.53	0.355	0.575	0.908
Spleen	1.31	1.72	1.20	1.40	1.42	1.57	0.07	0.304	0.668	0.556
Bursa	1.78	1.75	1.31	1.84	1.49	1.94	0.12	0.677	0.834	0.319
Small intestine	38.78	42.15	33.03	38.64	29.75	36.00	1.27	0.056	0.068	0.568
Cecum	2.39^b^	3.25^a^	2.96^ab^	3.11^ab^	2.55^ab^	2.86^ab^	0.087	0.026	0.815	0.036
Length (inches)										
Small intestine	64.63	67.75	62.88	61.48	63.00	64.43	0.83	0.355	0.330	0.361
Cecum	6.53	6.38	6.71	6.23	5.85	6.41	0.11	0.389	0.275	0.786

Data are presented as Means ± SEM. ^a-c^Different superscripts in a row differ significantly. SEM=Standard error of the pooled means

### Health biomarkers

Table 5 presents the effects of threonine supplementation during HS on serum health biomarkers in broilers. Total protein concentrations were lower (p = 0.006) in the HS and threonine-supplemented HS broilers than in the TN broilers, except in the HS-175 broilers. Likewise, HS reduced (p = 0.002) the albumin concentrations compared with the TN group. However, threonine supplementation at 125% and 175% during HS improved (p = 0.006) the albumin concentrations. The serum cholesterol level was higher (p < 0.001) in all experimental birds, except for the HS and HS-200 birds, compared with the TN birds. The LDL (p < 0.001) and LDL to HDL ratio (p < 0.001) were higher in all experimental groups, except for the HS-200 group, compared with the TN birds. (CORT) levels were higher (p < 0.001) in HS birds and significantly lower in HS-200 threonine-supplemented birds than in TN birds. However, serum metabolites, enzymes (except LDH in the HS-200 group), and hormones remained the same in all experimental groups. Regarding the serum oxidative status, the MDA level was elevated (p = 0.001), whereas SOD activity was reduced (p = 0.002) in HS birds compared with TN birds. Threonine supplementation reduced MDA and increased SOD activity compared with HS. The serum catalase levels were lower (p = 0.010) in the HS-150 and HS-200 threonine-containing broilers than in the TN broilers (Table 5).

**Table 5:** Effects of dietary threonine inclusion on serum health biomarkers of heat-stressed broilers.

Parameters	Treatment groups									

	TN	HS	HS-125	HS-150	HS-175	HS-200	SEM	p-value	Linear	Quadratic
Serum metabolites										
Total protein (g/dL)	6.00^a^	4.93^b^	4.68^b^	4.93^b^	5.13^ab^	4.94^b^	0.11	0.006	0.031	0.007
Albumin (g/dL)	2.19^a^	1.78^b^	1.92^ab^	1.87^b^	1.95^ab^	1.82^b^	0.03	0.002	0.017	0.060
Globulin (g/dL)	3.83	3.14	2.75	3.04	3.19	3.11	0.10	0.050	0.102	0.017
Cholesterol (mg/dL)	123^b^	134^ab^	142^a^	143^a^	142^a^	124^b^	2.04	<0.001	0.372	<0.001
Triglycerides (mg/dL)	196	176	188	186	192	196	2.29	0.064	0.266	0.022
HDL (mg/dL)	40.45	32.53	33.09	37.18	34.91	33.48	0.87	0.063	0.167	0.257
LDL (mg/dL)	43.06^c^	66.66^ab^	72.87^a^	68.99^a^	69.61^a^	50.94^bc^	2.22	<0.001	0.187	<0.001
LDL: HDL	1.10^b^	2.12^a^	2.27^a^	1.92^a^	2.00^a^	1.56^ab^	0.09	<0.001	0.248	<0.001
Creatinine (mg/dL)	0.33	0.27	0.28	0.24	0.24	0.23	0.02	0.749	0.152	0.689
BUN (mg/dL)	8.34^a^	8.07^a^	7.85^ab^	7.09^b^	8.45^a^	8.32^a^	0.11	0.001	0.833	0.002
Uric acid (mg/dL)	8.76	8.67	8.68	8.74	8.86	8.84	0.04	0.743	0.268	0.397
Enzymes										
ALT (U/L)	3.72	4.34	2.98	3.85	3.47	3.35	0.33	0.909	0.616	0.977
AST (U/L)	44.84	45.67	53.28	51.95	42.36	41.70	1.91	0.371	0.492	0.080
ALP (U/L)	373	369	387	414	370	436	23	0.953	0.486	0.854
LDH (U/L)	602^a^	724^a^	625^a^	542^ab^	538^ab^	413^b^	22	0.001	<0.001	0.043
CK (U/L)	33.64^ab^	55.20^a^	38.56^ab^	37.74^ab^	23.05^b^	31.98^ab^	2.87	0.033	0.057	0.354
Hormones										
fT3 (pg/mL)	6.95	6.24	6.02	5.54	5.61	5.62	0.24	0.546	0.079	0.397
fT4 (pg/mL)	18.85	17.50	17.89	18.66	17.94	17.65	0.21	0.363	0.372	0.853
Corticosterone (ng/mL)	16.51^bc^	22.49^a^	20.76^ab^	20.45^ab^	14.23^cd^	11.37^d^	0.71	<0.001	<0.001	<0.001
Redox status										
MDA (nmol/mL)	2.50^bc^	3.21^a^	2.91^ab^	2.81^abc^	2.19^c^	2.46^bc^	0.08	0.001	0.013	0.019
Catalase (U/mL)	13.56^a^	7.29^ab^	6.93^ab^	5.33^b^	9.15^ab^	5.01^b^	0.78	0.010	0.009	0.086
SOD (%)	30.69^a^	12.84^b^	36.68^a^	31.67^a^	34.90^a^	33.60^a^	1.96	0.002	0.032	0.992

Data are presented as Means ± SEM. ^a-d^Different superscripts in a row differ significantly. HDL=High-density lipoprotein, LDL=Low-density lipoprotein, BUN=Blood urea nitrogen, ALT=Alanine aminotransferase, AST=Aspartate aminotransferase, ALP=Alkaline phosphatase, LDH=Lactate dehydrogenase, CK=Creatine kinase, fT3=Free tri-iodothyronine, fT4=Free thyroxine, MDA=Malondialdehyde, SOD=Superoxide dismutase, SEM=Standard error of the pooled means

### Meat quality and oxidative status

The muscle pH at 45-min was significantly higher (p = 0.015) in the HS-150 broilers than in the HS broilers. Moreover, muscle crude protein was higher (p = 0.001) in TN birds than in HS and HS-200 birds. All the other parameters including pH_24hrs_, WHC, dry matter, crude fat, and ash contents remained unchanged among the experimental groups (Table 6). The pectoral muscle MDA was higher (p = 0.026) in HS birds than in TN birds, whereas 125% threonine supplementation reduced (p = 0.026) muscle MDA. Muscle catalase activities were lower (p = 0.042) in HS-175 threonine-supplemented birds compared with the HS birds. The activities of SOD in the pectoral muscle were lower (p = 0.011) in the HS- and HS-125 threonine-supplemented birds than in the TN birds (Table 6).

**Table 6:** Effects of dietary threonine inclusion on pectoral muscle physical characteristics, proximate analyses, and redox status in heat-stressed broilers.

Groups	Physical characteristics			Proximate analyses (%)				Redox status		
		
	pH 45 min	pH 24 h	WHC (%)	DM	CP	CF	Ash	MDA (nmol/mL)	Catalase (U/mL)	SOD (%)
TN	6.41^ab^	5.87	14.70	30.86	85.79^a^	4.26	6.16	1.88^b^	90^ab^	63^a^
HS	6.31^b^	5.58	15.85	28.29	83.31^b^	3.18	6.10	3.09^a^	112^a^	43^b^
HS-125	6.73^ab^	5.87	14.62	25.83	84.10^ab^	4.59	6.13	2.52^ab^	85^ab^	42^b^
HS-150	6.78^a^	5.87	15.87	29.65	82.68^b^	4.51	6.12	1.94^b^	83^ab^	49^ab^
HS-175	6.40^ab^	5.73	13.47	28.58	84.34^ab^	4.61	6.29	2.30^ab^	81^b^	57^ab^
HS-200	6.62^ab^	5.73	16.56	28.50	82.43^b^	2.99	6.08	2.16^ab^	85^ab^	52^ab^
SEM	0.05	0.05	0.63	1.01	0.27	0.25	0.11	0.12	3.12	1.98
p-value	0.015	0.457	0.771	0.826	0.001	0.214	0.997	0.026	0.042	0.011
Linear	0.138	0.817	0.795	0.742	0.003	0.670	0.947	0.481	0.049	0.806
Quadratic	0.119	0.840	0.731	0.446	0.216	0.162	0.943	0.212	0.894	0.008

Data are presented as Means ± SEM. ^a-b^Different superscripts in a column differ significantly. TN=Thermoneutral group, HS=Heat stress, WHC=Water holding capacity, DM=Dry matter, CP=Crude protein, CF=Crude fiber, MDA=Malondialdehyde, SOD=Superoxide dismutase, SEM=Standard error of the pooled means

### Jejunal histology and selected cells counting

Jejunal histology revealed that the VH (p = 0.016) and VSA (p = 0.039) were lower in the HS-125 group than in the HS-150 group. The other parameters remained unchanged in the experimental groups (Table 7). The population of acidic goblet cells was lower (p < 0.001) in HS birds, whereas they were higher (p < 0.001) in HS-175 and HS-200 threonine-supplemented birds compared with TN birds, respectively. The basic goblet cell counts were lower (p = 0.35) in the HS-150 birds compared with the HS birds. The number of total goblet cells was higher (p < 0.001) in the HS-175 and HS-200 broilers than in the other experimental groups. The IELs were elevated (p < 0.001) in the HS birds compared with the other experimental groups; however, they were lower (p < 0.001) in the HS-175 and HS-200 threonine-supplemented broilers compared with the other experimental groups (Table 8).

**Table 7:** Effects of dietary threonine inclusion on jejunal histology of heat-stressed broilers.

Groups	VH (µm)	VW (µm)	CD (µm)	VSA (mm^2^)	VH: CD
TN	1416	145^ab^	150	0.651^ab^	9.80
HS	1412	138^ab^	145	0.609^ab^	10.39
HS-125	1303	129^b^	144	0.527^b^	10.14
HS-150	1442	159^a^	150	0.718^a^	9.11
HS-175	1362	139^ab^	150	0.594^ab^	9.82
HS-200	1354	139^ab^	145	0.593^ab^	9.69
SEM	21	2.60	3.10	0.018	0.18
p-value	0.504	0.016	0.984	0.039	0.477
Linear	0.485	0.902	0.907	0.663	0.395
Quadratic	0.853	0.931	0.979	0.918	0.953

Data are presented as Means ± SEM. ^a-b^Different superscripts in a column differ significantly. TN=Thermoneutral group, HS=Heat stress, VH=Villus height, VW=Villus width, CD=Crypt depth, VSA=Villus surface area, SEM=Standard error of the pooled means

**Table 8:** Effects of dietary threonine inclusion on jejunal goblet cell and intraepithelial lymphocyte counts of heat-stressed broilers.

Groups	AGC (cells/villus)	BGC (cells/villus)	TGC (cells/villus)	IEL (Cells/100 µm)
TN	105^b^	39^ab^	144^b^	5.5^b^
HS	87^c^	41^a^	128^b^	14.0^a^
HS-125	95^bc^	38^ab^	133^b^	5.6^b^
HS-150	109^b^	29^b^	138^b^	5.6^b^
HS-175	141^a^	40^ab^	181^a^	4.5^c^
HS-200	151^a^	35^ab^	186^a^	4.6^c^
SEM	5	1	5	0.6
p-value	<0.001	0.035	<0.001	<0.001
Linear	<0.001	0.101	<0.001	<0.001
Quadratic	<0.001	0.381	<0.001	<0.001

Data are presented as Means ± SEM. ^a-c^Different superscripts in a column differ significantly. TN=Thermoneutral group, HS=Heat stress, AGC=Acidic goblet cells, BGC=Basic goblet cells, TGC=Total goblet cells, IEL=Intraepithelial lymphocytes, SEM=Standard error of the pooled means

## DISCUSSION

HS adversely affects the performance and health of the broilers. To mitigate the adverse effects of HS, various strategies are used. In a recent review, El-sabrout *et al*. [30] highlighted the significance of anti-stress feed additives and environmental enrichment to mitigate the adverse effects of stress on birds’ behavior, welfare, health, and productivity. The review concluded that incorporating anti-stress feed additives (vitamins, minerals, organic acids, and phytobiotics) and environmental enrichment is crucial for maintaining overall health and productivity in flocks during stressful events.

In this study, we supplemented HS broilers with high-dose threonine from days 22 to 42. Threonine was supplemented at 100%, 125%, 150%, 175%, and 200% of the current NRC recommendations. These percentages correspond to 7.51, 9.44, 11.27, 13.20, and 15.00 g of threonine per kilogram of feed, respectively.

### Productive performance

The growth performance of broilers is an important indicator of feed efficiency. In our experiment, the FI was reduced numerically, whereas the BWG was significantly lower in HS birds than in TN birds. These findings are consistent with those of a previous study by Ahmad *et al*. [31], which reported that during stress, animals adapt to reduce metabolic heat production by reducing their feed intake. Moreover, reductions in FI and BWG have been reported in previous studies by Mascarenhas *et al*. [3], and Sun *et al*. [4] of birds exposed to cyclic or chronic HS. The FI, body weights, and BWG were lower in all threonine-supplemented birds in HS during the fifth, sixth, and entire periods of HS. On the one hand, the reduced FI is due to HS, while it could also be due to the supplementation of high threonine levels. High dietary threonine levels modify the amino acid balance of the diet, and it might also change the plasma amino acid concentrations and balance. The brain senses variations in plasma amino acids and, in response, can reduce FI [32]. Contrary to our study, Debnath *et al*. [10] reported increased FI and BWG in broilers reared under subtropic conditions when supplemented with different concentrations of threonine. According to these authors, the observed increase in FI could be attributed to threonine-induced stimulation of thyroid activity, which in turn enhanced the metabolic rate and led to greater feed intake [33]. The difference between the findings of Debnath *et al*. [10] and our study may be due to differences in bird strain, HS period, and environmental factors. Similarly, Wasman [34] reported increased body weights and weight gains in threonine-supplemented broilers under HS. The FCR remained unchanged in the experimental birds, consistent with previous findings by Wasman [34] and Ghanima *et al*. [35].

### Viscera development

In our study, the effects on visceral weights indicated that HS significantly increased liver weight compared with TN, which might be due to inflammation of the liver, being sensitive to hypoxia, and the high temperature, as reported by Miao *et al*. [36]. However, in our study, threonine supplementation during HS reversed the liver weights in the range of TN birds’ liver weight, which shows that threonine might have improved the damage induced by HS, and this is similar to the findings of Ciftci and Ceylan [37], who stated that the relative liver weights were reduced by threonine supplementation in broiler chicken under normal management conditions. In this study, heart weight was significantly lower in threonine-supplemented HS birds than in TN birds. The decrease in heat weight could be attributed to decreased cell cycle activity and increased apoptosis under HS [38]. Furthermore, alteration in feed intake, absorption, and utilization of nutrients, along with depletion in fat reserve/storage and diversion of energy resources toward stress responses, could also account for the observed changes in the viscera weights [39]. The weights of the other studied organs remained unchanged and consistent with a previous study by Rezaeipour *et al*. [40], which reported that threonine supplementation did not influence the internal organ weights when administered in broilers.

### Health biomarkers

Serum protein levels indicate protein metabolism and immunity function. Avian total protein contains albumin and α, β, and γ-globulin. High total protein concentrations are associated with elevated levels of albumin and globulin. Our study showed that total protein concentrations were significantly lower in the HS group, and threonine supplementation did not show improvement in these parameters. Our findings contradict a previous report by Khan *et al*. [5], which reported that total protein concentrations remained unchanged in broilers under HS. The lower serum total protein levels observed in our study may be attributed to compromised liver function during HS in broilers, as serum albumin concentrations were also lower under these conditions. However, no changes in ALT and AST levels were observed during HS. The serum total cholesterol and LDL levels were higher in all experimental birds, except the HS-200 birds, compared with the TN birds; however, a numerical increase in these attributes was also observed in the HS birds.

Furthermore, the LDL to HDL ratio was also higher in all experimental groups, except in the threonine HS-200 group, compared with the TN group. An increase in blood cholesterol concentration [5] has been reported during HS, likely due to the mobilization of fat reserves under the influence of metabolic and hormonal disturbances, such as elevated circulating glucocorticoids [41]. Our results showed that higher threonine doses partially reduced total cholesterol and LDL concentrations in HS broilers. A previous study by Jiang *et al*. [42] reported that threonine regulates lipid metabolism and deposition, and its supplementation enhances hepatic lipid metabolism [43]. Another by Wang *et al*. [44] in humans found that threonine levels were linked to a lower risk of atherogenic lipid profiles.

CORT levels were higher in HS birds, indicating stress in birds exposed to high environmental temperatures. This finding is consistent with a previous study by El-Prollosy *et al*. [45], which reported that exposure to high temperatures during embryogenesis increased CORT levels in post-hatch roosters. In our study, the supplementation of threonine to HS birds, particularly at higher doses, reduced the levels of CORT, indicating the importance of threonine in lowering stress levels in birds. CORT is the primary glucocorticoid secreted in response to HS-induced activation of the hypothalamic-pituitary-adrenal cortex axis [46]. The role of CORT in resisting stress by mobilizing energy reserves was previously described by Khan *et al*. [5] and Xu *et al*. [47]. An increase in serum CK, although non-significant in the present study, reflected muscle damage and threonine supplementation reduced serum CK concentrations in HS birds, suggesting muscle-protecting effects. Furthermore, higher threonine concentrations also lower serum LDH levels in HS birds.

### Oxidative status

Reactive oxygen species (ROS) are naturally generated during cellular metabolism, but their production markedly increases under HS. When ROS levels exceed the antioxidant defense system, they oxidize cellular components such as DNA, lipid membranes, and proteins, leading to oxidative stress [48]. In our study, the oxidative status markers, including serum and muscle MDA and SOD, were perturbed under HS, and threonine supplementation reduced lipid peroxidation, as indicated by a reduction in MDA levels and increased SOD activities under HS. These results are consistent with previous findings by Hu *et al*. [49]. Min *et al*. [17] reported that threonine deficiency and/or excess significantly affected serum SOD activities but did not affect serum MDA. Conversely, Chen *et al*. [14] reported that higher dietary threonine concentrations decreased serum MDA levels. The authors suggested that enhanced oxidative status may be attributed to improved immunity, reduced pathogen colonization, and better intestinal integrity. In another study, Azzam *et al*. [50] reported that dietary threonine supplementation in laying hens improved their antioxidant status by enhancing SOD activity. Similarly, Debnath *et al*. [10] found that threonine supplementation enhanced SOD activity in broilers under HS. In the present study, the reduction in MDA levels may be linked to elevated SOD activity under the influence of threonine supplementation.

### Meat quality

HS during the growth period of broilers is associated with undesirable meat attributes and a loss of quality [51], which is an important consideration from the consumer’s point of view. In our study, the muscle pH at 45-min remained non-significant among all the treatment groups, but it was significantly different between the HS and HS-150 threonine groups. The pH of the comparison groups was acidic in the HS group. This could be due to anerobic glycolysis during HS in muscles, which is a characteristic feature of HS. Moreover, the muscle crude protein was significantly higher in TN birds than in HS and HS-200 birds. The lower crude protein content in the HS-200 threonine group may be due to an imbalance between amino acids due to higher threonine concentrations in the blood or meat of the birds. All other parameters, including the pH at 24-h, WHC, dry matter, crude fat, and ash content, remained unchanged.

### Jejunal histology and cell count

Attributes of jejunal histology, such as VW and VSA, were numerically higher only in threonine-supplemented HS-150 birds. It has been reported previously that approximately 30%–50% of threonine, along with several other amino acids, is directly utilized by the small intestine and is not accessible to other tissues outside of the intestine [15]. Consistent with our findings, Tanure *et al*. [52] observed no significant effects of different digestible threonine levels on VH, CD, and the ratio of these parameters in broilers during early age. Our study revealed that acidic goblet cells were significantly lower in HS birds, whereas threonine supplementation significantly increased acidic goblet cells. These findings are consistent with those of previous studies by Debnath *et al*. [10] and Nichols and Bertolo [53], indicating that threonine supplementation led to a notable increase in the number of goblet cells, thereby enhancing the protection of the intestines through mucin production. The elevation of threonine levels or the Thr/Lys ratio in the broiler diet resulted in a significant increase in the density of goblet cells in both the challenged and unchallenged groups of salmonella [54]. The IELs were significantly elevated in the HS birds compared with the other experimental groups. Interestingly, the HS-175 and HS-200 threonine-supplemented birds showed the lowest IEL counts compared with the other threonine-supplemented groups. Increased infiltration of IELs in the small intestine during stress in birds indicates the initiation of inflammatory processes [55], and increased infiltration of IELs during HS in broilers was reported earlier [56]. HS triggers inflammation by inducing systemic inflammatory cytokines, such as tumor necrosis factor-α and interleukin (IL)-2 [57], while also increasing the jejunal expression of lymphokines like IL-6 [58]. These inflammatory compounds can recruit IELs in the intestine during HS. Threonine supplementation modulates inflammatory factors in broilers [14], fish [59], and rats [60].

## CONCLUSION

This study investigated the effects of high dietary threonine supplementation on growth performance, physiological biomarkers, oxidative status, meat quality, and intestinal histology in broilers subjected to cyclic HS. The results demonstrated that HS significantly impaired growth performance, feed intake, and various physiological parameters. Threonine supplementation at higher levels (175% and 200% of NRC recommendations) improved oxidative balance by reducing serum and muscle MDA levels while enhancing SOD activity. In addition, threonine supplementation alleviated stress responses by lowering serum CORT levels and improving liver health markers. Intestinal histological analyses revealed that threonine supplementation enhanced acidic goblet cell counts and reduced intraepithelial lymphocyte infiltration, suggesting improved intestinal integrity. However, despite these physiological benefits, threonine supplementation did not improve body weight gain or feed conversion efficiency in HS broilers, limiting its potential as a growth promoter under HS conditions.

The strength of this study lies in its comprehensive assessment of multiple physiological and histological parameters to evaluate the impact of high-dose threonine supplementation. The well-controlled experimental design, including cyclic HS exposure and dose-dependent supplementation, provides valuable insights into the potential of threonine as a dietary intervention under HS conditions.

However, this study has a limitation as it did not assess potential interactions between threonine and other essential amino acids, which could influence nutrient utilization and growth responses.

For future research, further investigations should explore optimal threonine-to-lysine ratios and their interactive effects on broiler physiology under HS. Moreover, molecular-level studies could help elucidate the underlying mechanisms of threonine’s role in oxidative stress regulation and gut health improvement in broilers exposed to high environmental temperatures.

In conclusion, while high-dose threonine supplementation partially mitigates HS-induced physiological disruptions, its lack of impact on growth performance suggests that it may not be a standalone solution for improving productivity in HS broilers.

## AUTHORS’ CONTRIBUTIONS

AK, MAR, MAS, HR, and MSY: Conceived, designed, and coordinated the study. MAR and MSY: Managed resources for the experiment and sample analysis. HR and MSY: Supervised the trial. AK, MAR, and MSY: Feed formulation and analysis. AK, SB, AsK, HFR, and MAR: Conducted the experiment, collected data, analyzed samples, and entered data. MAS, HR, and MSY: Statistical analysis and interpretation of data. AK, HR, and MAR: Drafted the manuscript. MAS and MSY: Critically analyzed, edited, and finalized the manuscript. All authors have read and approved the final manuscript.
